# Anticipated discrimination in daily life: Predictors, stress appraisals, and responses

**DOI:** 10.1371/journal.pone.0344805

**Published:** 2026-04-02

**Authors:** Lydia Q. Ong, Megan W. Wolk, Anthony L. Burrow, Monika Lohani, Patrick L. Hill, Nancy L. Sin

**Affiliations:** 1 Department of Psychology, The University of British Columbia, Vancouver, British Columbia, Canada; 2 Edwin S.H. Leong Centre for Healthy Aging, The University of British Columbia, Vancouver, British Columbia, Canada; 3 Department of Psychological and Brain Sciences, Washington University in St. Louis, St. Louis, Missouri, United States of America; 4 Department of Psychology, Cornell University, Ithaca, New York, United States of America; 5 Department of Psychology, University of Utah, Salt Lake City, Utah, United States of America; University of West Florida, UNITED STATES OF AMERICA

## Abstract

A large body of literature details the deleterious effects of everyday discrimination on health, focusing on stress processes after discrimination occurs. In contrast, less work has investigated what occurs prior to encountering discrimination when a person expects it. Using a 10-day daily diary design, the current study examined predictors and outcomes of anticipated discrimination. Participants included 341 U.S. adults aged 19–74 years (29% racial minorities, 68% women). Multiple regression examined predictors of anticipated and reported discrimination. Further, two-level multilevel models evaluated anticipated discrimination predicting discrimination occurrence, appraisals, affect, and physical symptoms. Results showed that discrimination was anticipated on 21% of days; racial minorities and people with more prior exposure to discrimination anticipated more daily discrimination than White participants and those with lower prior exposure. People who anticipated discrimination more often than others reported more daily discrimination and perceived discrimination as more stressful but also perceived greater control over the events. They additionally had relatively larger upticks in physical symptoms on days when discrimination occurred—but no differences in discrimination-related affect—compared to people who anticipated discrimination less frequently. Within-persons, anticipating discrimination on a given day (versus not) was associated with greater likelihood of reporting discrimination occurred later that day and greater perceived stress severity, but no differences in perceived control, affect, or physical symptoms. In sum, anticipated discrimination was fairly common in daily life, and individual differences in anticipated discrimination were linked to more perceived daily discrimination, higher perceived stress severity, and more discrimination-related physical symptoms.

## Introduction

Everyday discrimination—the perception of unfair treatment based on membership in a social group (e.g., age, race/ethnicity, sexual orientation)—is a psychosocial stressor associated with poor long-term health and well-being [1]. Acute stress processes (e.g., stress responses) surrounding everyday discrimination are theorized to be the nexus by which discrimination impacts long-term health [2]. Research has largely focused on examining the impacts of discrimination on stress responses after it has already occurred [1]. By contrast, much less is known about stress processes surrounding daily anticipated discrimination. Research from the broader daily stress literature provides theoretical and empirical support for the importance of anticipatory stress processes [3]. Based on the prevalence of perceived discrimination in daily life [1] and initial evidence that daily discrimination is anticipated some of the time [4], more work is needed to investigate the correlates and consequences of anticipated discrimination. Using a 10-day daily diary design, this preregistered study investigated sociodemographic predictors of daily anticipated discrimination and whether anticipation is associated with stress appraisals and discrimination-related affect and physical symptoms.

### Anticipatory stress

Anticipation is a distinct part of the stress process that produces an anticipatory stress response, in which psychological responses (e.g., increases in negative affect) accompanied by physiological responses (e.g., activation of autonomic, neuroendocrine, and immune systems) take place, regardless of whether the event comes to pass [3,5]. Supporting evidence demonstrates that moments when a stressor was anticipated (versus moments without anticipated stressors) were associated with elevated negative affect in the hours that followed, independent of whether the stressor occurred [6,7]. In a study that assessed laboratory stressors and ambulatory assessment of cortisol, morning cortisol secretion was greater on the day of an anticipated laboratory stressor, providing evidence for a physiological response to anticipated demands later in the day [8].

Currently, very little empirical work in the field has examined naturally occurring anticipated discrimination in daily life. Rather, studies predominantly assess imagined discrimination, via measures that pose hypothetical scenarios (e.g., “Imagine…the alarm begins to sound, and a security guard comes over to investigate…”) [9,10], assess retrospective evaluations of vigilance for discrimination (e.g., “How often do you try to prepare for possible insults before leaving home?”) [11], or use experimental laboratory manipulation [12]. Evidence from these studies suggests that anticipating discrimination is associated with less favorable health, such as poorer self-reported sleep quality [9] and higher cardiovascular risk [10]. The relatively few studies using a within-person design have focused on proactive coping (i.e., coping efforts prior to anticipated discrimination) [13] rather than anticipation, with the exception of one study. Using a 30-day daily diary design, Belloir and colleagues [4] examined associations of daily anticipated discrimination with subjective and actigraphy-assessed sleep among sexual and gender minority people of color. They found that daily anticipated discrimination was associated with greater same-night sleep disturbances and sleep-related impairment on the following day [4]. More studies using daily diary designs are needed to further understand stress processes related to naturally occurring instances of anticipated discrimination (e.g., appraisals) and whether anticipation subsequently mitigates or exacerbates discrimination-related stress responses.

### Who anticipates discrimination?

The transdisciplinary model of stress asserts that greater cumulative stress exposure increases the expectation as well as perceived occurrence of stressors [5]. This is because the brain is believed to operate like a prediction machine, appraising events as threatening in part based on one’s history of stressor exposure [14]. Additionally, minority stress theory [15] and social safety theories [16–18] posit that experiences of stigma, prejudice, or discrimination signal to the nervous system that future threats are likely. Empirical work on stigma consciousness documented that individuals with higher expectations of being stereotyped by others were more likely to perceive discrimination directed toward their group and toward themselves [19]. Furthermore, individuals higher in stigma consciousness tended to be members of social groups which were historically stereotyped and discriminated against [19], underscoring the role of the broader social structure in contributing to expectations and reports of discrimination.

Due to systemic barriers and marginalization [2], reports of perceived discrimination are also patterned by sociodemographic factors. Racial/ethnic minorities often report more cumulative lifetime and everyday discrimination than White participants [20–22]. In support of this, research suggests that racial/ethnic minorities are more commonly socialized to prepare for and cope with discrimination [23]. In addition, among the most common perceived reasons for everyday discrimination is one’s age [24,25], and age discrimination is especially pertinent for older adults in the health and work domains [26]. In an experimental study, women and racial minorities anticipated more discrimination in a hypothetical workplace [27]. However, it remains unknown whether sociodemographic differences are evident for everyday experiences of anticipated discrimination.

### Appraisals of anticipated discrimination

In addition to the anticipatory stress response, anticipating discrimination may be associated with other stress processes, namely appraisals [5]. For instance, individuals appraise how much control they have over a stressor and how severe their stress experience is. An individual’s appraisals are associated with health, independent of stressor occurrence and “objective” ratings of stressor severity [28]. Appraisals of an anticipated stressor influence proactive coping strategies to mitigate the potential consequences [29]. Prior work showed that participants chose higher levels of active or problem-focused coping strategies (e.g., “make a plan of action and follow it”) to cope with anticipated stressors perceived to be under one’s personal control, compared to relatively uncontrollable stressors [30]. One study demonstrated that perceived control mitigated the link between discrimination and poor health among Black Americans [31]. Notably, a study investigating proactive coping found that 83% of an African American sample reported that they engaged in strategies to cope with racism before it occurred [13]. While theory and evidence suggest that “seeing it coming” confers benefits [32], proactive coping strategies for anticipated discrimination can also be highly effortful: For instance, concealing one’s stigmatized identity is cognitively, affectively, and behaviorally taxing and associated with negative psychological outcomes [33]. As such, anticipated discrimination could be associated with higher perceived control, yet higher perceived stress severity.

### Anticipated discrimination as a moderator of the stress response

Empirical evidence from the daily stress literature shows that, within-persons, anticipating a stressor is accompanied by increases in negative affect, providing evidence for the anticipatory stress response [34]. Whether anticipating discrimination on a given day subsequently moderates discrimination-related responses is less clear. Within the daily stress literature, two studies found that anticipating a stressor to occur in the next few hours did not buffer associations between stressor occurrence and elevated negative affect [6,34]. The authors speculated that anticipation may not be beneficial due to perseverative cognitions such as worry [6]. However, two other daily stress studies have found evidence for anticipation as a stress buffer. Among young adults, anticipating an at-home stressor to occur in the next 24 hours was associated with smaller upticks in negative affect when the stressor occurred, compared to when the stressor was not anticipated [7]. Additionally, anticipating stressors to occur within the day buffered the relationship between perceived stress levels and elevated negative affect [35].

Individual differences in anticipation may also moderate discrimination stress responses. On the one hand, people who tend to experience heightened anticipation are theorized to have stress responses that are less favorable for health [5]. This general tendency toward heightened anticipation is characterized by vigilance—the propensity to attend to and protect against anticipated discrimination by monitoring and/or modifying one’s behavior—which is associated with higher perceived stress and poorer health among Black Americans [11,36,37]. On the other hand, previous work demonstrated that racial minorities displayed attenuated stress responses to discrimination compared to White individuals [25,38,39]. This counterintuitive pattern may potentially be due to several reasons. First, parents might engage in racial/ethnic socialization, whereby they raise their children’s awareness of bias and prepare them to cope with discrimination [23]. Second, vicarious experiences of racism in one’s social network could aid in the acquired development of skilled responses (i.e., knowing whether and how to respond) [40]. Lastly, initial evidence documents high rates of proactive coping among African Americans [13]. A coordinated analysis of two daily diary studies found that proactive coping buffered the association between daily stressors and physical health in young adults, such that those with high trait proactive coping reported relatively fewer physical health symptoms and better self-rated health [41].

### Aims of the current study

The primary aims of the current study were to examine sociodemographic predictors of daily anticipated discrimination, and whether anticipation was related to stress appraisals and discrimination-related stress responses. Anticipated discrimination was captured using a 10-day daily diary design in a racially/ethnically heterogenous sample of 341 adults in the United States. We hypothesized that:

- (1) People who identified as a racial/ethnic minority, a woman or gender minority, were older, or had higher baseline reports of everyday discrimination will report relatively greater anticipated daily discrimination and discrimination occurrence.
- (2a) People who anticipate more daily discrimination on average will report more discrimination occurrence.
- (2b) Anticipating discrimination on a given day will be associated with higher likelihood of discrimination occurrence as well as higher perceived stress severity and higher perceived control.
- (3a) On days when a person anticipated discrimination to occur later that day, there will be a smaller stress response to discrimination occurrence (i.e., smaller downticks in positive affect and smaller upticks in negative affect and physical symptoms), compared to days without prior anticipation.
- (3b) Individuals who anticipated discrimination more frequently than others will have a smaller stress response to discrimination occurrence (i.e., higher positive affect, lower negative affect, and fewer physical symptoms), compared to individuals who do not anticipate discrimination as often.

## Method

### Participants

Participants were recruited as part of a broader online study using SurveySignal panels from April 15 to June 21, 2021, in which they completed a baseline questionnaire, 10 weekdays of morning and evening surveys, and a follow-up questionnaire [42]. To be eligible, participants had to be at least 18 years old and living in the U.S. All participants provided written informed consent and approval was obtained by the institutional review board at Washington University in St. Louis (#202009095). Of the 583 participants who joined the study, SurveySignal retained 354 participants who completed at least five days of daily diaries. Participants were also excluded from the current analyses if they had missing data for the Everyday Discrimination Scale (n = 4), sociodemographics (n = 4), depressive symptoms (n = 11), or outcome variables (n = 3; some participants were missing multiple variables). The final analytic sample consisted of 341 participants and 3410 days of data. Participant characteristics are displayed in Table 1.

**Table 1:** Participant characteristics.

Variable	Mean (*SD*) or *N* (%)
Participant characteristics	
Age, years	39.16 (12.75)
Gender	
Woman	231 (67.8%)
Man	98 (28.7%)
Transgender, genderqueer, or prefer not to answer	12 (3.5%)
Race/ethnicity	
White	242 (71.1%)
Asian American/Pacific Islander	33 (9.7%)
Black	31 (9.0%)
Biracial/Multiracial	16 (4.7%)
Hispanic/LatinX	13 (3.8%)
Native American	4 (1.1%)
Prefer not to answer	2 (0.6%)
Education (has bachelor’s degree)	249 (73.0%)
Depressive symptoms, 0–60 scale	15.82 (12.16)
Everyday Discrimination Scale, 1–6 scale	2.13 (0.95)
Daily diary variables	
Anticipated discrimination	706 days (20.7%)
Discrimination occurrence	123 days (3.6%)
Perceived stress severity, 0–3 scale	1.58 (0.85)
Perceived control, 0–3 scale	0.84 (0.82)
Negative affect, 1–5 scale	1.98 (0.80)
Positive affect, 1–5 scale	3.34 (0.84)
Number of physical symptoms, 0–14 checklist	1.16 (1.32)

SD = Standard Deviation.

### Measures

#### Baseline everyday discrimination.

Participants completed the Everyday Discrimination Scale [43] in the baseline questionnaire, which consisted of 9 items inquiring how often people encountered various types of unfair treatment in their day-to-day lives (e.g., “You are treated with less courtesy than other people are”; “You are threatened or harassed”). Responses options were (1) Never, (2) Less than once a year, (3) A few times a year, (4) A few times a month, (5) At least once a week, and (6) Almost everyday*.* Scores were averaged such that higher scores reflected a greater frequency of baseline everyday discrimination.

#### Daily anticipated discrimination.

In the morning surveys, participants were asked, “Today, how frequently do you expect the following events to occur?” (Never, Rarely, Sometimes, Often). Events were six common daily stressors from the Daily Inventory of Stressful Events [44], including “discrimination.” In line with previous work [7], we adapted the measure’s opening stem question to capture the expectation of events, rather than their occurrence. Anticipated discrimination was coded as 0 (Never) and 1 (Rarely, Sometimes, or Often)*.* Person-means for anticipated discrimination were calculated by averaging scores across the 10 days and reflected the proportion of study days on which discrimination was anticipated.

#### Daily discrimination occurrence.

In the evening surveys, participants were asked, “Which of the following types of stressors did you experience today?” (Yes, No) and provided with a list of the same six stressors as the morning surveys, including “discrimination” [44]. Discrimination occurrence was coded as 0 (No) and 1 (Yes). Person-means for discrimination occurrence were calculated by averaging scores across the 10 days and reflected the proportion of study days on which discrimination occurred.

#### Perceived control and stress severity.

If participants indicated that discrimination had occurred, they were further asked, “How much control did you have over the event from (0) None at all to (3) A lot?” as well as “How stressed did you feel on a scale from (0) None at all to (3) A lot?” Higher scores reflected higher perceived control and stress severity.

#### Daily affect.

Daily affect was assessed using 20 items from the Positive and Negative Affect Schedule [45] and six additional items capturing low-arousal emotions (e.g., relaxed, peaceful, down) [46]. Daily positive affect was assessed with 13 positive emotions (e.g., interested, excited, enthusiastic) and daily negative affect was assessed with 13 negative emotions (e.g., distressed, upset, scared). Participants rated to what extent they felt each emotion that day, ranging from (1) Very slightly or not at all to (5) Extremely. Daily positive and negative affect were calculated by taking the average ratings across the respective items each day.

#### Daily physical symptoms.

Physical symptoms were measured with a checklist of 13 items adapted from Cohen and Hoberman [47]. The original measure was shortened to reduce participant burden when applied to a daily diary design. We opted to omit symptoms that are relatively rarer day-to-day (e.g., blurred vision, pulled ligament) or less applicable to inquire at the daily level (e.g., weight change of 5 lbs or more). Participants were asked which of the following symptoms they experienced each day: headache, backache, muscle soreness, fatigue, cough, sore throat, fever, other cold/flu symptoms, nausea, poor appetite, chest pain, dizziness, shortness of breath, other physical symptoms, or none*.* Affirmative responses were summed to compute the number of physical symptoms each day [39].

#### Covariates.

All covariates were measured in the baseline questionnaire. Covariates related to discrimination and/or affect were included [48]: age (continuous), gender (coded as man, woman, or other—inclusive of transgender, genderqueer, or any additionally specified gender), race/ethnicity (coded as White, Black, Asian, or other—inclusive of Native American, Native Hawaiian, Hispanic or Latinx, and multiracial), education (dummy coded as less than bachelor’s degree vs. bachelor’s degree or higher), and depressive symptoms assessed via the 20-item Center for Epidemiological Studies Depression Scale and summed into a continuous variable [49]. Racial/ethnic or gender categories were analytically combined in instances where the individual sample sizes were very small.

### Analytic approach

Analyses were preregistered on the Open Science Framework and can be found here: https://osf.io/k59eu/?view_only=ef9abeb95b00473a937f768c34485d58. To test Hypothesis 1, two multiple linear regression models predicted the 10-day average frequency of anticipated discrimination and discrimination occurrence, separately. Focal predictors included average prior exposure to everyday discrimination, race/ethnicity, gender, and age. Depressive symptoms and education level were included as covariates.

For Hypotheses 2 and 3, multilevel models with restricted maximum likelihood estimation (*lme4* and *lmerTest* packages in *R* [50]) were used to account for the nesting of days (Level 1) within persons (Level 2). To test Hypothesis 2, three models evaluated anticipated discrimination as a predictor of daily discrimination occurrence, perceived control, and perceived stress severity. To test Hypothesis 3, three models evaluated anticipated discrimination and discrimination occurrence as predictors of daily negative affect, positive affect, and physical symptoms. Two interactions were included: Anticipated Discrimination (between-person) x Discrimination Occurrence (within-person) and Anticipated Discrimination (within-person) x Discrimination Occurrence (within-person). In all multilevel models, Level 1 predictors were person-mean centered and continuous Level 2 predictors were grand-mean centered. Person-means for anticipated discrimination as well as person-level covariates were entered at Level 2. A random slope for anticipated discrimination (within-person) was included to allow individuals to differ from one another in the associations of anticipated discrimination with same-day outcomes; this random slope was dropped in cases where the model had a singular fit warning [51].

## Results

### Sample descriptives

Across all diary days (n_days_ = 3410), discrimination was anticipated on 20.7% of days (n_days_ = 706), while discrimination occurrence was reported on 3.6% of days (n_days_ = 123). Of the discrimination days, 63.4% were anticipated. Participants typically appraised low perceived control over discrimination (*M* = 0.8, 0–3 scale) and moderate perceived stress severity (*M* = 1.6, 0–3 scale). On average, positive affect was somewhat high (*M* = 3.3, 1–5 scale), negative affect was lower (*M* = 2.0), and there was just over one physical symptom per day (Table 1). The intraclass correlation coefficients indicated that 54% of the variation in daily anticipated discrimination, 28% in discrimination occurrence, 38% in negative affect, 31% in positive affect, and 35% in physical symptoms were attributable to between-person differences.

### Predictors of anticipated discrimination and occurrence

In line with Hypothesis 1, compared to White participants and people with lower baseline everyday discrimination, racial/ethnic minorities and people with higher baseline everyday discrimination (i.e., more prior exposure to discrimination on average) anticipated daily discrimination more frequently over the 10-day period (racial/ethnic minorities: b ranging from 0.21–0.25, 95% *CI* ranges [0.11–0.35]; baseline everyday discrimination: b = 0.15, 95% *CI* [0.11–0.19]). However, compared to men, women and gender minorities did not differ in anticipated daily discrimination (b ranging from −0.03–0.04, 95% *CI* ranges [−0.12–0.21]). Furthermore, there were no differences in anticipated discrimination based on age, education level, or depressive symptoms (b ranging from 0.00–0.03, 95% *CI* ranges [−0.04–0.10]) (Table 2).

**Table 2:** Baseline everyday discrimination and sociodemographic factors predicting 10-day average daily anticipated discrimination and occurrence.

	Anticipated Discrimination			Discrimination Occurrence		
Predictor	Est.	95% *CI*	*p*	Est.	95% *CI*	*p*
Intercept	−0.18	−0.33 – −0.03	**0.017**	−0.01	−0.06–0.04	0.808
Baseline Everyday Discrimination	0.15	0.11–0.19	**<0.001**	0.03	0.02–0.04	**<0.001**
Asian or Pacific Islander (vs. White)	0.21	0.11–0.31	**<0.001**	0.00	−0.03–0.04	0.959
Black (vs. White)	0.22	0.12–0.32	**<0.001**	0.00	−0.03–0.04	0.812
Other races (vs. White)	0.25	0.16–0.35	**<0.001**	0.04	0.00–0.07	**0.032**
Other gender (vs. Man)	0.04	−0.12–0.21	0.592	−0.03	−0.08–0.03	0.331
Woman (vs. Man)	−0.03	−0.09–0.04	0.400	−0.01	−0.03–0.01	0.359
Age	0.00	−0.00–0.00	0.993	−0.00	−0.00–0.00	0.272
Education (1 = bachelor’s degree)	0.03	−0.04–0.10	0.374	−0.02	−0.04–0.01	0.163
Depressive symptoms	0.00	−0.00–0.00	0.769	0.00	−0.00–0.00	0.081

*N* = 341 persons (3410 days). Estimates represent unstandardized regression coefficients. Continuous variables were centered. In the models, “Other races” comprised of Native American, Native Hawaiian, Hispanic or Latinx, and multiracial. CI = confidence interval.

Although they anticipated more daily discrimination, participants who identified as Black or Asian/Pacific Islander did not differ in their reports of daily discrimination occurrence compared to White participants (b = 0.00, 95% *CI* [−0.03–0.04] for both groups). Those identifying with other races/ethnicities (consisting of Native American, Native Hawaiian, Hispanic or Latinx, or bi/multiracial) reported significantly more daily discrimination than White participants (b = 0.04, 95% *CI* [0.00–0.07]). Additionally, people with higher baseline everyday discrimination subsequently reported more daily discrimination (b = 0.03, 95% *CI* [0.02–0.04]). There were no differences in daily discrimination occurrence based on age, gender, education level, or depressive symptoms (b ranging from −0.03–0.00, 95% *CI* ranges [−0.08–0.03]) (Table 2).

### Anticipated discrimination predicting perceived control and stress severity

When controlling for all covariates, results from multilevel models provided support for Hypothesis 2a. On average, people who anticipated daily discrimination more frequently also reported higher daily discrimination occurrence (b = 0.14, 95% *CI* [0.11–0.17]). They perceived discrimination to be greater in stress severity (b = 0.34, 95% *CI* [0.26–0.41]) but also perceived greater control (b = 0.16, 95% *CI* [0.10–0.22]), compared to people who anticipated discrimination less frequently. In partial support of Hypothesis 2b, within-persons, anticipating discrimination on a given day was significantly associated with reporting discrimination later that day (b = 0.02, 95% *CI* [0.00–0.05]). However, within-person anticipated discrimination was associated with higher perceived stress severity (b = 0.05, 95% *CI* [0.01–0.10]), and was not associated with perceived control over the discrimination event (b = 0.00, 95% *CI* [−0.03–0.03]) (Table 3).

**Table 3:** Daily anticipated discrimination predicting discrimination occurrence, perceived stress severity, and perceived control over discrimination events.

	Discrimination Occurrence			Perceived Stress Severity			Perceived Control		
Predictor	Est.	95% *CI*	*p*	Est.	95% *CI*	*p*	Est.	95% *CI*	*p*
Fixed effects									
Intercept	0.05	0.01–0.08	**0.004**	0.13	0.06–0.20	**<0.001**	0.10	0.04–0.15	**<0.001**
Age	−0.00	−0.00–0.00	0.117	−0.00	−0.00 – −0.00	**0.031**	−0.00	−0.00–0.00	0.209
Other gender (vs. Man)	−0.04	−0.10–0.01	0.145	−0.10	−0.23–0.02	0.108	−0.10	−0.20 – −0.00	**0.040**
Woman (vs. Man)	−0.01	−0.03–0.01	0.485	−0.03	−0.08–0.02	0.220	−0.03	−0.07–0.00	0.071
Asian American/ Pacific Islander (vs. White)	−0.03	−0.06–0.01	0.121	−0.09	−0.17 – −0.01	**0.026**	−0.05	−0.11–0.01	0.116
Black (vs. White)	−0.02	−0.06–0.02	0.285	−0.04	−0.12–0.04	0.351	−0.02	−0.09–0.04	0.443
Other race (vs. White)	0.01	−0.03–0.04	0.762	0.03	−0.04–0.11	0.376	−0.01	−0.07–0.05	0.686
Education (1 = bachelor’s degree)	−0.03	−0.05 – −0.00	**0.028**	−0.08	−0.14 – −0.03	**0.001**	−0.06	−0.10 – −0.02	**0.002**
Depressive symptoms	0.00	0.00–0.00	**0.017**	0.00	−0.00–0.00	0.184	0.00	−0.00–0.00	0.156
Study day	0.00	−0.00–0.00	0.489	0.00	−0.00–0.01	0.334	0.00	−0.00–0.00	0.685
Anticipated discrimination (BP)	0.14	0.11–0.17	**<0.001**	0.34	0.26–0.41	**<0.001**	0.16	0.10–0.22	**<0.001**
Anticipated discrimination (WP)	0.02	0.00–0.05	**0.042**	0.05	0.01–0.10	**0.013**	0.00	−0.03–0.03	0.975
Random effects (variance)									
Residual	0.03			0.10			0.06		
Intercept	0.01			0.03			0.02		
Slope for anticipated discrimination (WP)	0.00			0.01			0.00		
Correlation: Intercept, Anticipated Discrimination (WP)	0.44			0.56			−0.51		

*N* = 341 persons (3410 days). Estimates represent unstandardized regression coefficients. Continuous variables were centered. CI = confidence interval. BP = between-person. WP = within-person.

### Anticipated discrimination and discrimination occurrence predicting affect and physical symptoms

Between-persons, individuals who anticipated discrimination to occur more frequently had higher average negative affect (b = 0.52, 95% *CI* [0.36–0.68]), positive affect (b = 0.48, 95% *CI* [0.20–0.77), and physical symptoms (b = 0.77, 95% *CI* [0.30–1.24], compared to those with less frequent expectations for discrimination. Within-persons, anticipating daily discrimination did not significantly predict same-day affect or physical symptoms (b ranging from 0.03–0.16, 95% *CI* ranges [−0.07–0.33]). Additionally, discrimination occurrence was significantly associated with negative affect (but not positive affect or physical symptoms) at the between- and within-person levels. People who reported more daily discrimination on average had higher negative affect (b = 0.62, 95% *CI* [0.12–1.13]). Within-persons, days on which discrimination occurred were associated with higher same-day negative affect (b = 0.17, 95% *CI* [0.06–0.29]) (Table 4).

**Table 4:** Anticipated discrimination, occurrence, and their interaction predicting daily negative affect, positive affect, and physical symptoms.

	Negative Affect			Positive Affect			Physical Symptoms		
Predictor	Est.	95% *CI*	*p*	Est.	95% *CI*	*p*	Est.	95% *CI*	*p*
Fixed effects									
Intercept	1.34	1.20–1.47	**<0.001**	3.52	3.29–3.75	**<0.001**	0.91	0.53–1.30	**<0.001**
Age	−0.00	−0.01 – −0.00	**0.013**	0.01	0.00–0.01	**0.035**	0.01	0.00–0.02	**0.008**
Other gender (vs. Man)	−0.20	−0.45–0.04	0.104	−0.49	−0.93 – −0.06	**0.025**	0.61	−0.10–1.33	0.091
Woman (vs. Man)	−0.07	−0.17–0.02	0.121	−0.14	−0.31–0.03	0.105	0.17	−0.11–0.44	0.237
Asian or Pacific Islander (vs. White)	−0.19	−0.35 – −0.04	**0.014**	−0.41	−0.69 – −0.14	**0.003**	−0.49	−0.94 – −0.04	**0.033**
Black (vs. White)	−0.16	−0.32 – −0.00	**0.046**	0.17	−0.11–0.45	0.227	−0.45	−0.91–0.01	0.055
Other race (vs. White)	−0.22	−0.36 – −0.07	**0.004**	−0.06	−0.32–0.20	0.642	−0.00	−0.43–0.43	0.988
Education (1 = bachelor’s degree)	0.05	−0.05–0.15	0.338	−0.07	−0.25–0.10	0.416	−0.24	−0.52–0.05	0.108
Depressive symptoms	0.02	0.02–0.03	**<0.001**	−0.03	−0.04 – −0.02	**<0.001**	0.04	0.03–0.05	**<0.001**
Study day	−0.00	−0.01–0.00	0.061	−0.02	−0.03 – −0.01	**<0.001**	−0.04	−0.05 – −0.02	**<0.001**
Anticipated discrimination (BP)	0.52	0.36–0.68	**<0.001**	0.48	0.20–0.77	**0.001**	0.77	0.30–1.24	**0.001**
Anticipated discrimination (WP)	0.03	−0.05–0.11	0.432	0.02	−0.07–0.11	0.625	0.16	0.00–0.33	0.050
Discrimination occurrence (BP)	0.62	0.12–1.13	**0.016**	0.49	−0.40–1.38	0.277	1.06	−0.40–2.52	0.154
Discrimination occurrence (WP)	0.17	0.06–0.29	**0.003**	−0.02	−0.19–0.15	0.815	−0.13	−0.55–0.29	0.550
Anticipated discrimination (BP) x Discrimination occurrence (WP)	0.10	−0.16–0.35	0.461	−0.13	−0.50–0.24	0.507	1.32	0.41–2.23	**0.005**
Anticipated discrimination (WP) x Discrimination occurrence (WP)	−0.07	−0.36–0.22	0.640	0.04	−0.34–0.42	0.830	−0.02	−0.85–0.82	0.971
Random effects (variance)									
Residual	0.16			0.26			1.12		
Intercept	0.14			0.47			1.23		
Slope for discrimination occurrence (WP)	0.02			0.11			1.11		
Slope for anticipated discrimination (WP)	0.11			0.11			0.27		
Correlation: Intercept, Discrimination occurrence (WP)	0.27			−0.19			0.14		
Correlation: Intercept, Discrimination anticipation (WP)	−0.17			−0.18			0.21		

*N* = 341 persons (3410 days). Estimates represent unstandardized regression coefficients. Continuous variables were centered. BP = between-person; WP = within-person.

**Fig 1:**
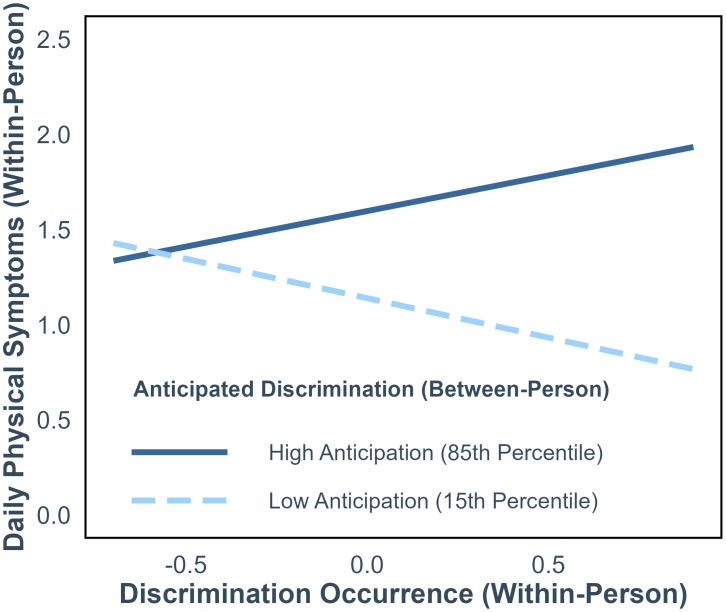
Average frequency of anticipated discrimination interacts with daily discrimination occurrence to predict same-day physical symptoms. People who anticipated discrimination more frequently had greater upticks in physical symptoms on days when discrimination occurred (simple slope: b = 0.37, *SE* = 0.18, *p* = .047), compared to people who anticipated discrimination less frequently (simple slope: b = −0.41, *SE* = 0.28, *p* = .15).

Neither between- nor within-person anticipated discrimination moderated the within-person relationships between daily discrimination occurrence and positive affect (b ranging from −0.13–0.04, 95% *CI* ranges [−0.50–0.43]) or negative affect (b ranging from −0.07–0.10, 95% *CI* ranges [−0.36–0.35]), providing no evidence for Hypothesis 3a (Table 4). However, anticipated discrimination (between-person) moderated the within-person relationship of daily discrimination occurrence with physical symptoms, but in the opposite direction than was predicted in Hypothesis 3b (b = 1.32, 95% *CI* [0.41–2.23]). As depicted in Fig 1, on days when discrimination occurred, individuals who tended to anticipate discrimination more frequently showed a greater uptick in physical symptoms, compared to individuals who anticipated discrimination less frequently.

## Discussion

Using a 10-day daily diary design, the present study examined predictors of anticipated daily discrimination and associations of anticipation with discrimination-related stress appraisals and responses. Among 341 U.S. adults, we found that racial/ethnic minorities and individuals with greater prior exposure to discrimination anticipated relatively more daily discrimination. On average, people who anticipated daily discrimination more often reported relatively more daily discrimination events and found discrimination to be more stressful, but felt they had more control over the discrimination events. Lastly, on days when self-reported discrimination took place, these individuals also displayed a relatively larger increase in the physical symptoms they experienced.

### Who anticipated and reported daily discrimination?

We found partial support for Hypothesis 1, such that people with higher scores on the Everyday Discrimination Scale at baseline (i.e., reported more prior exposure to discrimination) subsequently anticipated and reported more daily discrimination. This aligns with multiple theoretical frameworks, such as minority stress theory which asserts that stigmatized individuals learn to anticipate discrimination and rejection [15]. Prior exposure to stigma or social marginalization is theorized to lead to anticipated danger and social unsafety [17]. The resulting vigilance and environmental monitoring [17] could partly contribute to greater perceptions of discrimination. Furthermore, results support broader stress models suggesting that past experiences shape expectations for and reports of future events [5].

Contrary to our hypothesis, there were few demographic differences in anticipated discrimination and occurrence. There were no age, gender, or educational differences in daily anticipated discrimination or occurrence. Although all racial/ethnic minority groups anticipated more daily discrimination than White participants, only those who were Native American, Native Hawaiian, Hispanic/Latinx, or bi/multiracial reported greater discrimination occurrence over the 10-day study period. This could suggest underlying racial/ethnic differences in how our measure was interpreted and what types of events meet the threshold for “discrimination.” In addition, daily discrimination was reported on 3.6% of days (n_days_ = 123), limiting statistical power. Alternatively, results could reflect the influence of intersectionality, whereby participants’ various social identities (e.g., race, gender, age) intersect to shape their experiences of discrimination [52]. This could potentially explain why few differences were observed when making comparisons based on a single category at a time.

### Was anticipating discrimination linked to same-day experiences?

Anticipating discrimination on a given day was not associated with daily affect or physical symptoms, which contrasts previous empirical evidence documenting a within-person link between anticipating daily stressors and higher negative affect [34], conceptualized as the anticipatory stress response [3]. Unlike Scott and colleagues [34], in the present study, affect was assessed in the evening surveys whereas anticipated discrimination was reported in the morning surveys. Thus, the gap between assessments may have been too large to capture the anticipatory stress response. Future studies with multiple assessments per day could provide greater granularity for capturing the anticipatory stress response to discrimination as it unfolds.

In line with our predictions, anticipating discrimination in the morning was associated with a higher likelihood of reporting discrimination occurrence later in the evening survey. However, contrary to Hypotheses 2b and 3a, “seeing it coming” did not confer benefits for stress appraisals or responses if discrimination occurred. On days when discrimination occurred, prior anticipation was associated with appraising the discrimination event as more stressful, compared to when discrimination occurred but was not anticipated. Yet, there were no differences in perceived control over the discrimination events. A potential explanation is perseverative cognition, which is a repetitive activation of the cognitive representation of an upcoming or not immediately present stressor [53]. According to the perseverative cognition hypothesis, these repetitive thoughts cause maintained activation of physiological systems, prolong the experience of stress, and lead to poorer health in the long-term [53]. This further aligns with findings that, among sexual and gender minority people of color, anticipated discrimination was associated with same-night sleep disturbances and next-day sleep-related impairment [4]. Scholars have previously posed that anticipatory stress (particularly racism-related stress) engenders perseverative cognition, resulting in high levels of anticipation being harmful rather than beneficial [54].

Furthermore, while days with discrimination were associated with higher same-day negative affect, anticipating discrimination did not buffer this relationship. This null interaction effect is consistent with two prior daily stress studies [6,34] but inconsistent with one that found a buffering effect for at-home stressors among young adults [7]. The disparity in findings may be due to differences in stressor domains because different types of anticipated stressors have been associated with different coping strategies [7]. Importantly, in previous work as well as in the current study, there was no follow-up assessing whether the reported stressors were consistent with what participants anticipated beforehand. In instances where there were incongruencies between the expected event and the event that occurred, prior coping efforts (if any took place) may have been unsuccessful at mitigating the negative affective stress response. Alternatively, it is possible that participants did not anticipate specific discriminatory events to take place but rather reported general expectations for discrimination. Future daily diary studies can investigate these nuances in anticipated discrimination and occurrence, as well as whether anticipation predicts the use of beneficial proactive coping strategies for buffering the stress response.

### Did people who anticipated discrimination more than others have different daily experiences?

At the between-person level, participants who anticipated daily discrimination more often than others reported relatively more discrimination events over the 10 days. Greater levels of anticipated discrimination appear to reflect vigilance, or the heightened tendency to attend to potential threats of discrimination that are not immediately present [11]. In line with the literature on racism-related vigilance [37], individuals who anticipated more daily discrimination than others had higher average negative affect and physical symptoms. Unexpectedly, they also experienced higher average positive affect. A potential reason is that greater rates of anticipated discrimination and discrimination occurrence could reflect greater social engagement overall, which would expose individuals to more positive as well as negative social encounters. Previous work demonstrated that people who reported more daily stressors also had more daily positive events, which are associated with higher positive affect [55]. Future research can examine these other aspects of daily events among individuals who anticipate discrimination, and whether daily positive events buffer the link between anticipated discrimination and same-day outcomes.

High levels of anticipated discrimination were associated with stress appraisals and exacerbated stress responses. First, participants who anticipated relatively more daily discrimination appraised discrimination events to be more, rather than less, stressful. Additionally, they experienced greater upticks in physical symptoms on days discrimination occurred, compared to people who anticipated discrimination less often. These findings support the transdisciplinary model of stress [5] by showing that greater anticipated discrimination between-persons poses a vulnerability for acute stress responses. Interestingly, when discrimination did occur, they reported greater perceived control over the discrimination events compared to those with lower levels of anticipation. Vigilance for discrimination may result in constant preparation that results in greater perceived control, but these efforts in and of themselves can cause stress, such as concealing a stigmatized identity [33]. Another possible explanation is that individuals who tend to anticipate more discrimination than others face discrimination events that are more severe and threatening, compared to the discrimination experienced by people with lower levels of anticipation.

Interestingly, participants with greater levels of anticipated discrimination did not differ from their counterparts in discrimination-related positive or negative affect, although they showed a greater physical symptom response. This suggests that discrimination-related physical symptoms reflect a response that is distinct from affective experience, rather than a byproduct of elevated negative affect. While previous research has largely focused on affective correlates of daily discrimination [1], assessing physical symptoms as well can provide a more comprehensive understanding of the health correlates of both anticipated and reported discrimination. Habitual anticipation may become embodied via daily physical symptoms to influence long-term health [56]. This raises avenues for future research to examine whether individuals who frequently anticipate discrimination exhibit “skin-deep resilience,” whereby psychological resilience comes at a cost to physical health [57].

### Strengths and limitations

This study contributes to the nascent literature on within-person daily anticipated discrimination and complements the broader body of work on between-person differences in anticipated discrimination, vigilance, and health-related outcomes. A key strength includes the repeated measurement design, which enabled us to capture naturally occurring instances of daily anticipated discrimination and stress processes as they unfold within-persons. To our knowledge, this is the first study to assess daily anticipated discrimination in the mornings prior to assessing daily occurrences of discrimination, which enabled us to better test whether anticipating discrimination subsequently moderated discrimination-related affect or physical symptoms.

A few limitations should be noted. Although anticipated discrimination was fairly common (20.7% of days), the low rates of daily discrimination occurrence (3.6% of days) limited our statistical power for detecting within-person interactions between anticipated discrimination and discrimination occurrence. Future studies with heterogenous samples may benefit from longer daily diary assessment periods, multiple assessment points per day, and including weekends. Relatedly, our sample was not sufficiently powered to further probe certain sociodemographic characteristics (e.g., there were very few gender diverse participants) and we did not assess all relevant factors in the baseline questionnaire (e.g., disability). In addition, the single-item daily discrimination measure may have led to underreporting of perceived discrimination. In two U.S. national surveys, Grollman and Hagiwara [58] found that measures inquiring about “discrimination” yielded lower reports compared to measures inquiring about “unfair treatment.” It is possible that the unfair treatment participants experience sometimes is not considered severe enough to meet their threshold for “discrimination,” even if the experience is attributed to one’s social identity [58]. Furthermore, we did not assess follow-up details on the reported daily discrimination, such as perceived reasons for the experience (e.g., because of one’s age) or whether the perceived discrimination that occurred aligned with the discrimination that participants had anticipated. As such, we cannot deduce whether there was true concordance between morning reports of anticipated discrimination and evening reports of discrimination occurrence, which warrants further investigation into anticipation as a stress buffering or exacerbating factor. The current study sets the stage for future research to investigate the different types of daily anticipated discrimination (e.g., racial/ethnic discrimination) and whether stress appraisals and responses differ between the various types.

## Conclusion

In sum, findings from this 10-day daily diary study indicated that racial/ethnic minorities and people with more prior exposure to discrimination anticipated more discrimination in their daily lives. Anticipating discrimination on a given day predicted greater likelihood of discrimination occurrence, but anticipation was not protective for stress responses and, in fact, resulted in feeling greater perceived stress severity. People who anticipated discrimination more often than others perceived greater stress severity, greater control over the discrimination events, and displayed larger physical symptom responses to discrimination. Taken together, the results paint a picture of cumulative exposure to everyday discrimination leading to a habitual process of anticipation, resulting in greater perceived stress severity, perceived control, and exacerbated physical symptom responses to discrimination. Future research is encouraged to investigate whether proactive coping for daily discrimination mitigates stress responses.

## Supporting information

S1 Appendix Full measures for baseline everyday discrimination, daily anticipated discrimination, daily discrimination occurrence, perceived control and stress severity, daily affect, and daily physical symptoms. (PDF)
