# Supplementary material for: Anticipated discrimination in daily life: Predictors, stress appraisals, and responses
Source: PLoS One. 2026 Apr 2;21(4):e0344805. doi: 10.1371/journal.pone.0344805 (PMC13046157; doi:10.1371/journal.pone.0344805)
Supplement: S1 Appendix — (PDF) [file pone.0344805.s001.pdf]

S1 Appendix. Full measures for baseline everyday discrimination, daily anticipated discrimination, daily discrimination occurrence, perceived control and stress severity, daily affect, and daily physical symptoms.

Everyday Discrimination Scale (Williams et al., 1997)

In your day-to-day life, how often do any of the following things happen to you?

	Never (1)	Less than once a year (2)	A few times a year (3)	A few times a month (4)	At least once a week (5)	Almost everyday (6)
You are treated with less courtesy than other people are. (1)						
You are treated with less respect than other people are. (2)						
You receive poorer service than other people at restaurants or stores. (3)						
People act as if they think you are not smart. (4)						
People act as if they are afraid of you. (5)						
People act as if they						

think you are dishonest. (6)
People act as if they're better than you are. (7)
You are called names or insulted. (8)
You are threatened or harassed. (9)

If you reported any of the experiences above, what do you think is the main reason (or reasons) for these experiences?
Your Ancestry or National Origins (1)
Your Gender (2)
Your Race (3)
Your Age (4)
Your Religion (5)
Your Height (6)
Another Aspect of Your Physical Appearance (7)
Your Sexual Orientation (8)
Your Education or Income Level (9)
A Physical Disability (10)
Other (11) ____

Expectations for the Day (Adapted from Almeida et al., 2002; Sin & Almeida, 2018)

Today, how frequently do you expect the following events to occur?

	Never (1)	Rarely (2)	Sometimes (3)	Often (4)
Argument or disagreement with anyone (1)				
Work or school related stressor (2)				
Home related stressor (3)				
Discrimination (4)				
Close friend or relative stressor (5)				
Anything else that people would consider stressful (6)				
Positive social interaction, in-person (7)				
Positive social interaction, remote (such as on the phone or online) (8)				
Positive event at work or school (9)				
Positive volunteer or service experience (10)				
Positive event at home (11)				
Positive event that happened to a close friend or family member (12)				
Spent time enjoying or viewing nature (13)				

Other positive event (14)

Daily Stressor Occurrence (Almeida et al., 2002)

Which of the following types of stressors did you experience today? If they occurred, rate how stressful the event was and how much control you had over the event from 0 (None at all) to 3 (A lot).

	Did this event happen?		IF YES - How stressed did you feel, on a scale from 0 to 3?				IF YES - How much control did you have over the event from 0 to 3?			
	Yes (1)	No (2)	0 (1)	1 (2)	2 (3)	3 (4)	0 (1)	1 (2)	2 (3)	3 (4)
Argument or disagreement with anyone (1)										
Work or school related event (2)										
Home related event (3)										
Discrimination (4)										
Something happened to close friend or relative event (5)										
Anything else that people would consider stressful (6)										

Affect (Posner, Russell, & Peterson, 2005; Watson & Clark, 1999)

This scale consists of several words that describe different feelings and emotions. Read each item and then indicate to what extent you felt this way **today**:

	Very slightly or not at all (1)	A little (2)	Moderately (3)	Quite a bit (4)	Extremely (5)
Interested (1)					
Distressed (2)					
Excited (3)					
Upset (4)					
Strong (5)					
Guilty (6)					
Scared (7)					
Hostile (8)					
Enthusiastic (9)					
Proud (10)					
Irritable (11)					
Alert (12)					
Ashamed (13)					
Inspired (14)					
Nervous (15)					
Determined (16)					
Attentive (17)					
Jittery (18)					
Active (19)					
Afraid (20)					
Calm (21)					
Peaceful (22)					
Sad (23)					

Down (24)

Relaxed (25)

Lethargic (26)

Physical Symptoms (Cohen & Hoberman, 1983)

Which of the following did you experience today? (Check all that apply)

- ☐ Headache (1)
- ☐ Backache (2)
- ☐ Muscle soreness (3)
- ☐ Fatigue (4)
- ☐ Cough (5)
- ☐ Sore throat (6)
- ☐ Fever (7)
- ☐ Other cold or flu symptoms (9)
- ☐ Nausea (33)
- ☐ Poor appetite (34)
- ☐ Chest pain (10)
- ☐ Dizziness (11)
- ☐ Other physical symptoms (35)
